# Global burden of pulmonary arterial hypertension and health inequality in women of childbearing age from 1990 to 2021, with projections up to 2040: the global burden of disease 2021 study

**DOI:** 10.3389/fpubh.2025.1700855

**Published:** 2025-11-18

**Authors:** Letai Li, Haiyan Wang, Yutong Chen, Xinlin Tan, Ting Gao, Nanting Chen, Xinyue Hu, Rui Liu, Ling Zhang, Yingjiu Jiang, Mengjun Bie, Jiajie Leng

**Affiliations:** 1 Department of Cardiothoracic Surgery, The First Affiliated Hospital of Chongqing Medical University, Chongqing, China; 2 First Clinical Medical College, The First Clinical Chongqing Medical University, Chongqing, China; 3 West China School of Medicine, Sichuan University, Chengdu, Sichuan, China; 4 Department of Anatomy, Laboratory of Neuroscience and Tissue Engineering, Basic Medical College, Chongqing Medical University, Chongqing, China

**Keywords:** pulmonary arterial hypertension, global burden of diseases, five sociodemographic index regions, national epidemiology, women of childbearing age

## Abstract

**Objective:**

To assess the global burden, trends, and inequalities of pulmonary arterial hypertension (PAH) among women of childbearing age (WCBA) from 1990 to 2021, projecting future trends to 2040.

**Methods:**

Using Global Burden of Disease (GBD) 2021 data, we analyzed PAH mortality, incidence, prevalence, and disability-adjusted life years (DALYs) among WCBA aged 15 to 49. Trends were assessed via age-standardized rates (ASR), estimated annual percentage change (EAPC), joinpoint regression, decomposition, and inequality analyses. Future burden was projected using Bayesian age-period-cohort, auto-regressive moving average, and exponential smoothing models.

**Results:**

The absolute number of PAH cases among WCBA increased globally from 1990 to 2021. Critically, the global age-standardized incidence rate (ASIR) showed a neglected upward trend (EAPC = 0.12), while mortality and DALY rates declined. Significant inequalities were observed, with the ASIR highest in low-middle SDI regions and prevalence highest in high SDI regions. Decomposition analysis identified population growth as the primary driver of increasing burden in low-resource settings. Projections indicate a continued rise in incidence by 2040, alongside declining mortality.

**Conclusion:**

This study demonstrated a persistent and growing PAH burden among WCBA, marked by significant inequalities. The rising incidence, particularly in low-resource settings, coupled with the cumulative prevalence in high-income regions, underscores an urgent need for targeted public health actions. Region-specific interventions are vital, including integrating PAH screening into maternal health programs in resource-poor settings and optimizing long-term management in high-income countries.

## Introduction

Pulmonary arterial hypertension (PAH) represents a significant global health burden. In women of childbearing age (WCBA), PAH is defined as a mean pulmonary arterial pressure (mPAP) greater than 20 mmHg at rest (measured by cardiac catheterization in patients for over 3 months) and a pulmonary vascular resistance (PVR) of ≥3 Wood units (WU) (1). Notably, PAH is a leading cause of death among WCBA, posing devastating risks to maternal survival and fetal outcomes during pregnancy. PAH can develop from a common and serious complication of congenital heart disease known as Eisenmenger syndrome, which is also the leading cause of death from PAH in WCBA. Consequently, to safeguard women’s public health rights, there is an urgent need for clinical attention to the burden of PAH in WCBA.

The global prevalence of PAH was estimated at 1% in 2016, rising to 10% in those over 65 (2). However, data (3) specifically addressing PAH in WCBA remain scarce. With evolving diagnostic criteria, current epidemiological information for WCBA is fragmented and underreported, failing to reflect global trends. Recent guidelines, such as the 2022 European Society of Cardiology (ESC)/European Respiratory Society (ERS) guidelines, have refined diagnostic and treatment algorithms, yet their impact on the epidemiology of WCBA remains unclear (4). Despite PAH registries in regions such as Australia, Canada, New Zealand, and selected African countries, comprehensive, updated studies on reproductive-aged women are urgently needed (5–8), as addressing this gap is crucial for guiding targeted prevention and management.

High disease burdens often arise from underdeveloped sociodemographics and inadequate healthcare systems (9), and PAH is no exception. Approximately 80% of PAH patients live in developing countries (10), however, international guidelines lack sufficient focus on these regions. A JACC expert consensus highlights that low- and middle-income areas with high PAH incidence face late care access, heavy medication burdens, scarce resources, and hard-to-manage comorbidities (11, 12). It emphasizes that current guidelines are primarily based on high-income studies, underscoring the necessity to align them with global epidemiological realities.

Despite advances in global PAH epidemiology, critical gaps persist for vulnerable groups like WCBA (13). A 2022 Global Burden of Disease (GBD) systematic review identified 65 studies on PAH prevalence, incidence, and 1-year survival across 37 countries (1991–2021) (14), and a 2023 JACC GBD study explored the global PAH burden (15). A recent WHO report on non-communicable diseases and gender also highlights the paucity of sex-disaggregated data for conditions like PAH (16). However, studies focusing on WCBA-specific epidemiological characteristics, burden, and outcomes remain notably lacking. Given their unique physiological factors—including pregnancy-related hemodynamic stress and female hormonal influences—scarce systematic evidence limits understanding and hinders targeted strategies.

To address these shortcomings, we utilized GBD 2021 data to comprehensively assess PAH burden, trends, and inequalities in WCBA. Our objectives are: (1) descriptive analysis of PAH epidemiology in WCBA globally, across five Sociodemographic Index (SDI) regions, and in 204 countries/territories; (2) trend and decomposition analyses based on demographic and epidemiologic factors; (3) predictive analysis of global PAH burden in WCBA through 2040.

## Methods

### Data sources and definitions

This study utilized the GBD database, a comprehensive repository of global health and disease-related data. Developed in collaboration with the World Health Organization and other entities, the globe has been divided into 21 geographic regions, primarily based on geographic location, cultural similarities, and population health patterns. The GBD database contains a wide array of information on global epidemiology, the burden of disease, and health system performance from 1990 to the present. The detailed methodology, data resources, and modeling approaches applied in GBD have been fully explained elsewhere (17–19). The period 1990–2021 was chosen as it encompasses an era of significant change in global health policy, diagnostic criteria for PAH, and allows for the assessment of long-term trends and the impact of various interventions over time. In our study, the estimates and their 95% uncertainty intervals (UIs) for deaths, incidence, prevalence, and disability-adjusted life years (DALYs) of PAH in WCBA (aged 15–49) were extracted from GBD 2021 (20, 21). Currently, PAH is recognized as the most common type of PH in WCBA, and according to the modified World Health Organization (mWHO) classification of maternal cardiovascular risk, it is known to carry a very high risk of development. Incidence reflects the frequency with which a disease spreads within a specific population group, while prevalence refers to the overall burden of the disease. DALYs measure the total burden of disease—both from years of life lost due to premature death and years lived with a disability (22). All age-standardized rates (ASR) were calculated using the GBD world population standard to eliminate the influence of differences in age structure, allowing for comparability across regions and over time, and were reported per 100,000 population (23). By analyzing these four factors together, we can assess the impact of PAH in WCBA on patients and society as a whole, identify key inflection points in trends, and explore potential factors influencing these changes. Additionally, the Sociodemographic Index (SDI), which represents a composite measure of income, education, and fertility conditions and quantifies the level of socio-demographic development of a country or region, was employed, categorized into five levels based on the five SDI quintiles (i.e., low, medium-low, medium, medium-high, and high) (24). Focusing on global trends, five different SDI regions, and all the countries, this analysis aims to provide health policy insights on PAH in WCBA based on the findings, contributing to overall health development globally.

### Descriptive analysis

We conducted descriptive analyses globally, across all countries, and within five distinct SDI regions (high, medium-high, medium, medium-low, and low) in an effort to explore the burdens and trends of PAH in WCBA. The results depicted the global number of cases and ASR of deaths, incidence, prevalence, and DALYs of PAH in WCBA from 1990 to 2021. Additionally, data on the number of cases and ASR of deaths, incidence, prevalence, and DALYs for females in the five SDI regions and all countries for the years 1990 and 2021 have been included.

### Trend analysis

To further explore the spatial and temporal trends of PAH among WCBA and to develop precise and dynamic prevention policies, the present study conducted a multidimensional analysis to capture PAH trends globally, across five different SDI regions, and within 21 GBD regions. The estimated annual percentage change (EAPC) value and its 95% confidence interval (CI) were included to reflect the temporal trends in the burden of PAH from 1990 to 2021. These metrics were calculated using a log-linear regression: ln (ASR) = *α* + *β*x, where x = calendar year, and EAPC = (exp(β)–1) * 100% (25). The 95% CI was generated based on the standard errors produced by the log-linear regression. The ASR was considered to have increased if both the EAPC and its 95% CI were greater than 0; it was classified as decreased if they were less than 0. Otherwise, the rate was regarded as showing no significant change (26).

Subsequently, joinpoint regression analysis (Joinpoint Trend Analysis Software, 2022) was employed to divide the overall trend into multiple sub-segments based on the inflection points and compute the annual percentage change (APC) and average annual percentage change (AAPC), along with APC/AAPC estimates, to identify localized trends in the burden of PAH in WCBA and validate the (EAPC) results (25). The APC and its 95% CI were utilized to further assess the magnitude of the local epidemiologic trend, and the AAPC, a comprehensive and compact measure of the trend in the burden of pulmonary hypertension in WCBA over fixed intervals, was calculated as the weighted mean of the APC. If the lower limit of both the APC/AAPC estimate and its 95% CI is greater than 0, an upward trend is considered to have occurred during that period. Conversely, if both the APC/AAPC estimate and the upper bound of its 95% CI are less than 0, the trend is viewed as downward within that period. Otherwise, the trend is deemed stable (27, 28).

### Age-period-cohort analysis

The present study utilized the age-period-cohort (APC) model to assess the independent effects of age, period, and cohort on PAH incidence, prevalence, mortality, and age-specific rates of disability-adjusted life years. The model distinguishes between the roles of three temporal dimensions: the age effect, which reflects changes in biological and social dimensions with age; the period effect, arising from external factors such as environmental changes and health interventions; and the cohort effect, which captures the unique events or exposures experienced by a specific birth cohort over time. The modeling time span for this study was from 1990 to 2021. Relative risks (RRs) were calculated for each period and cohort, with RR values greater than 1 indicating an increased risk of incidence, prevalence, mortality, or age-standardized rates of disability-adjusted life years for PAH in WCBA. The statistical modeling and analysis was conducted utilizing R software (version 4.4.2), which was employed for the generation of visualizations. In this study, statistical significance was defined as *p*-values less than 0.05. The APC modeling was performed using online tools provided by the US National Cancer Institute, which is based on the log-linear Poisson model. The mathematical expression for this model is: lnErij = lnθijNij = *μ* + αi + βj + γk. In this equation, Erij represents the expected incidence rate, μ is the mean effect value, and αi, βj, and γk correspond to the amount of age, period, and cohort effects, respectively. The estimation uncertainty was resolved by calculating 95% uncertainty intervals (UI) through simulation plotting.

### Relationship between SDI and ASR

The relationship between SDI and ASR, i.e., Spearman’s correlation coefficient (r), is calculated as: *r* = 1–(6Σd_i^2^) / [n (n^2^–1)]; where d_i is the ordinal difference of the pairwise variables and n is the total number of observations. This is an important analytical dimension used in public health and epidemiological studies to assess the association between a society’s level of development and the burden of a particular disease (or health indicator). This analytical dimension is of particular significance in the fields of public health and epidemiology, as it facilitates the assessment of the correlation between a society’s demographic development level and the prevalence of specific diseases (or health indicators). SDI, a composite indicator reflecting income level, education level and fertility status of a country or region, has been shown to quantify the overall level of socio-demographic development. Conversely, ASR, an age-standardized rate, has been demonstrated as a means to eliminate the influence of differences in the age structure of different populations in the assessment of the burden of disease, thereby enabling the comparability of health indicators across regions and over time. The following text is intended to provide a comprehensive overview of the subject matter. The present study sought to elucidate the correlation between sociodemographic indices (SDI) and age-standardized rates (ASR) of PAH in WCBA across diverse geographical regions and national boundaries. To this end, Spearman’s correlation coefficients (r) were computed.

### Cross-country inequality analysis

In order to analyze inequalities in the burden of PAH in WCBA across countries, two standardized indicators were introduced in this study: the Slope Inequality Index (SII) and the Concentration Index (CI). The SII is calculated by fitting a regression line between the health indicator and the socioeconomic rank (based on SDI) of populations. It represents the absolute difference in the health indicator between the most and least advantaged. A positive SII indicates a higher burden among the more advantaged (higher SDI), while a negative SII indicates a higher burden among the less advantaged (lower SDI). The magnitude of the SII reflects the size of the absolute inequality.

The CI is derived from the concentration curve, which plots the cumulative proportion of the health indicator against the cumulative proportion of the population ranked by SDI. The CI is defined as twice the area between the concentration curve and the line of equality. It is computed as: CI = (2/*μ*) * *Σ*[i = 1 to n] f_i * y_i * R_i–1, where μ is the mean of the health variable, f_i is the population share of the i-th group, y_i is the health variable value for the i-th group, and R_i is the relative rank of the i-th group. The CI ranges from −1 to 1. A positive CI (pro-rich) indicates the health variable is concentrated among higher SDI groups, while a negative CI (pro-poor) indicates concentration among lower SDI groups. A value of zero represents perfect equality. The further the value is from zero, the greater the degree of inequality. As an absolute measure of health inequality, the slope inequality index is utilized to quantify the linear association between health indicators and socioeconomic status (SES). For specific calculations, the crude rates of incidence, prevalence, mortality, and disability-adjusted life years (DALYs) of peripheral artery disease (PAD) in each country are analyzed by correlating them with scales constructed based on sociodemographic indices (SDIs). A positive value of SII (>0) is indicative of a more favorable health outcome for individuals in the higher socioeconomic status group, while a negative value of SII (<0) is indicative of a more favorable health outcome for individuals in the lower socioeconomic status group. It is evident that the larger the absolute value of SII, the more pronounced the group differences in the health indicators become. Conversely, the concentration index constitutes a relative measurement tool, the calculation of which is achieved by integrating the area under the Lorenz concentration curve (16). The value of this index ranges from −1 to 1, with values closer to 0 indicating less inequality. When the concentration index (CI) is positive (CI > 0), it is indicative of superior health outcomes for wealthier groups; conversely, when the index is negative (CI < 0), it signifies enhanced health outcomes for poorer groups.

### Predictive analysis

To project the future trends of the PAH in WCBA from 2022 to 2040, we applied three methods: bayesian age-period-cohort (BAPC), auto-regressive moving average (ARIMA), and exponential smoothing (ETS) model (29). We adopted the BAPC model with integrated nested Laplace approximations (INLA) to analyze historical data and estimate PAH in WCBA burden. Vague, non-informative priors were used for all model parameters to minimize prior influence. For the ARIMA model, the parameters (p,d,q) were determined based on the autocorrelation function (ACF) and partial autocorrelation function (PACF) plots of the training data, and stationarity was ensured using the Augmented Dickey-Fuller test. The specific (p,d,q) orders for the final models are reported in the Supplementary material. For the ETS model, the error, trend, and seasonality components were selected automatically based on the Akaike Information Criterion (AIC). Model selection for final projection was based on the lowest Mean Absolute Error (MAE), Mean Absolute Percentage Error (MAPE), and Root Mean Square Error (RMSE) on the test set. All predictive analyses were conducted in R (version 4.4.2) using the bsts, forecast, and INLA packages. The sample size for modeling was the entire GBD 2021 time series for each outcome from 1990 to 2021. Furthermore, the uncertainty from the GBD data (95% UIs) was propagated through the predictive models by performing multiple simulations drawing from the distribution of the input ASR estimates, and the resulting prediction intervals reflect this propagated uncertainty. Sensitivity analyses were conducted by varying model parameters and training/test splits, which confirmed the robustness of our main conclusions. We adopted the BAPC model with integrated nested Laplace approximations to analyze historical data and estimate PAH in WCBA burden. ARIMA combines autoregressive and moving average models. It assumes data series are time-dependent random variables whose autocorrelation can predict future values based on past values (30). The ETS model forecasts by weighted averaging historical data, assigning higher weights to recent data. Exponential smoothing fits models by combining error, trend, and seasonality components via addition, multiplication, or no operation (31). We split the dataset into a 70% training set and 30% test set. After fitting the three models on the training data, we tested them on the test set. Using mean absolute error (MAE), mean absolute percentage error (MAPE), and root mean square error (RMSE) as performance metrics, we aimed to identify the optimal model (32).

### Decomposition analysis

Finally, this study used decomposition analysis to explore the drivers of changes in global PAH in WCBA deaths, incidence, prevalence, and DALYs from 1990 to 2021. Decomposition analysis allows assessment of the impact of three factors—population, aging and epidemiologic change—on the burden of PAH in WCBA. Decomposition analysis in this study was performed using the method proposed by Gupta. We disaggregated incidence, prevalence, deaths, and DALYs globally and across the 5 SDI regions. Detailed equations and procedures are in a previous publication (33).

## Results

### Descriptive analysis of PAH burden at global, regional, and national levels

**Figure 1:**
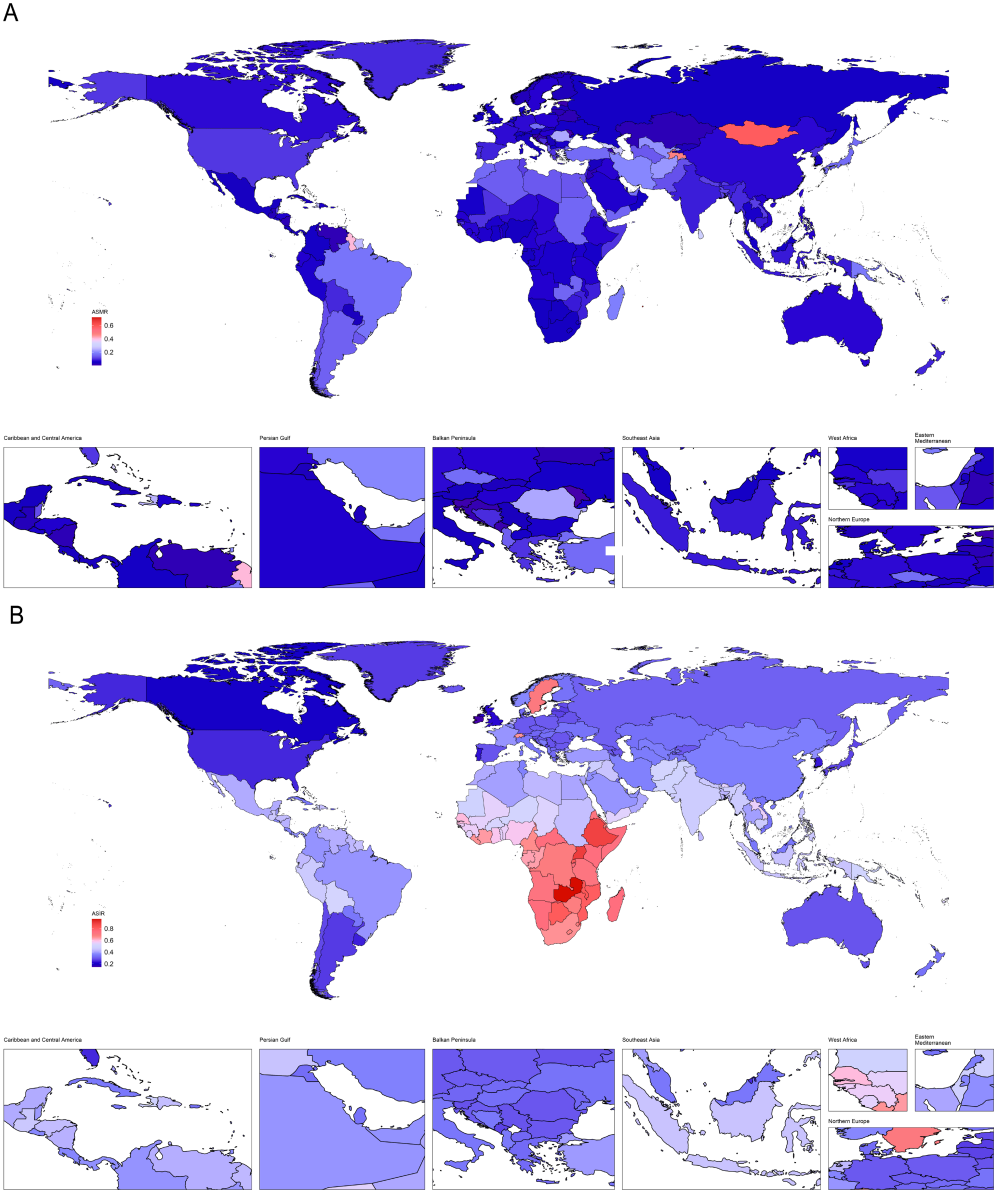
Descriptive analysis of PAH burden at national levels. **(A)** The ASR of deaths in 2021; **(B)** The ASR of incidence in 2021.

**Figure 2:**
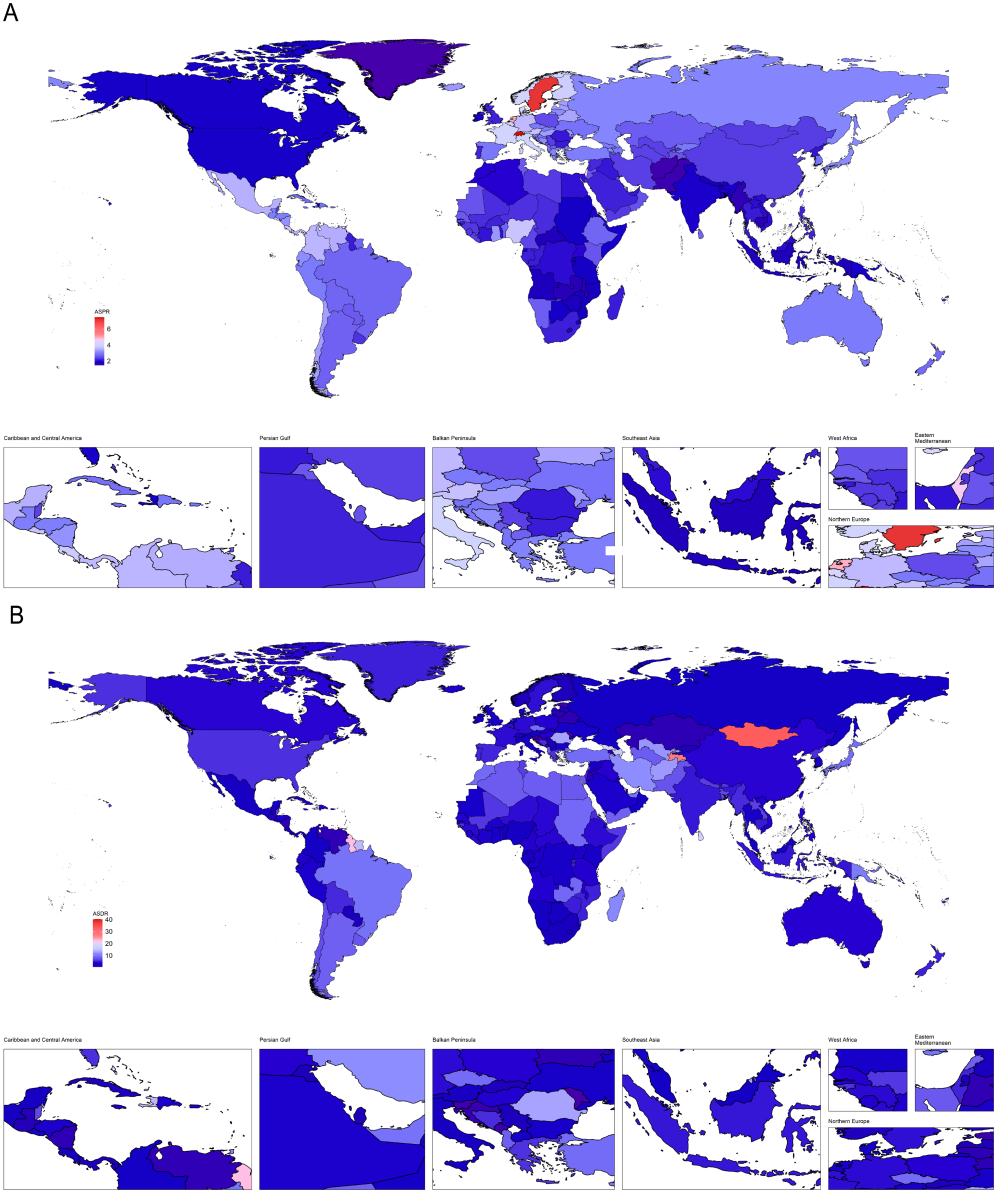
Descriptive analysis of PAH burden at national levels. **(A)** The ASR of prevalence in 2021; **(B)** The ASR of DALYs in 2021.

**Figure 3:**
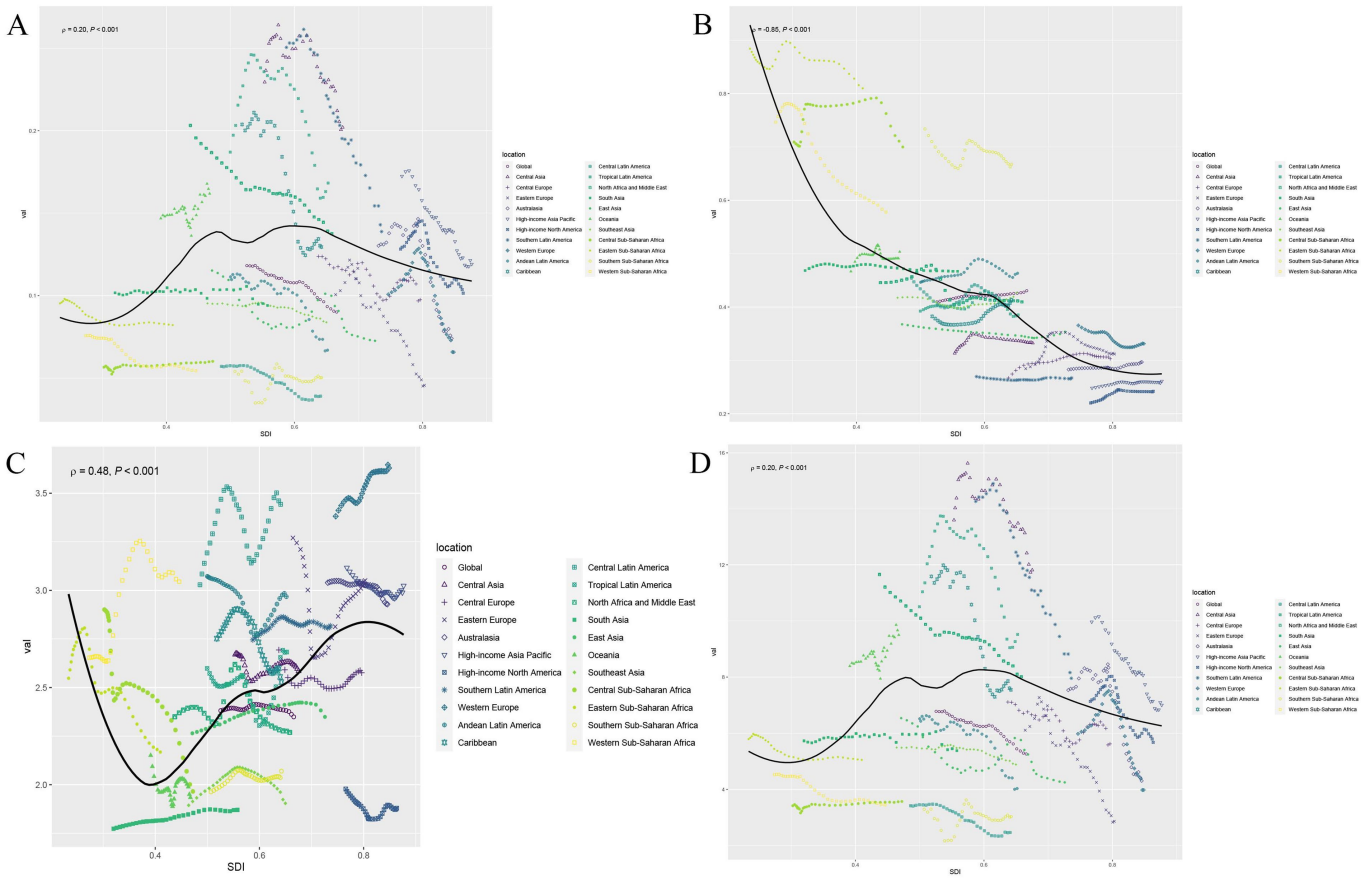
The ASR of deaths, incidence, prevalence, and DALYs in relation to SDI in different regions. **(A)** Deaths. **(B)** Incidence. **(C)** Prevalence. **(D)** DALYs. The dotted lines represent the different regions labeled in the legend, while the black curves indicate the overall trend. The *ρ* values and *p* values in the upper left corner of the subgraph represent the correlation coefficient and statistical significance, respectively.

Globally, the number of deaths, DALYs, incidence, and prevalence cases of PAH exhibited a significant upward trend from 1990 to 2021. At the same time, we analyzed the ASR values of PAH deaths, DALYs, incidence, and prevalence worldwide in 2021 at the national level, with results illustrated in Figures 1, 2 as a world map. However, compared to 1990, the ASR values in 2021 all decreased (Tables 1A–D). Consistent with the global trend, five SDI regions demonstrated a parallel tendency regarding the number of prevalence cases during the same period (1990–2021). Nevertheless, differences in the number of deaths, incidence, and DALYs exist among the five SDI regions compared to global averages. The high SDI and high-middle SDI regions showed a downward trend from 1990 to 2021, while the low SDI, low-middle SDI, and middle SDI regions continued to reflect trends similar to the global situation (Tables 1A–D; Supplementary Table 1). Notably, while differences between the five SDI regions persist, some significant patterns are evident. In 2021, the ASR value for prevalence of PAH was lowest in low-SDI regions and increased with higher SDI levels (Table 1C). Regarding incidence, the ASR values were highest in low-SDI regions and decreased as SDI levels increased (Table 1D; Figure 3). However, the highest ASR values for deaths and DALYs related to PAH were observed in the low-middle SDI region [ASMR (95% UIs): 0.10 (0.07–0.15); ASDR (95% UIs): 5.85 (4.13–8.32); Tables 1A,B].

**Table 1:** The number, ASR, and EAPC of deaths, DALYs, prevalence and incidence of PAH in 1990 and 2021 at global and regional levels: **(A)** DALYs. **(B)** Deaths. **(C)** Prevalence. **(D)** Incidence.

Regions	1990		2021		EAPC (95%CI)1990–2021
	Number (95%UIs)	ASR (95%UIs)	Number (95%UIs)	ASR (95%UIs)	
(A)					
Global	88386.82 (124333.95–61249.41)	6.78 (9.51–4.72)	103151.35 (137784.65–82299.47)	5.26 (7.04–4.19)	−0.76 (−0.84 to −0.67)
SDI quintiles					
High SDI	16003.6 (17824.84–14217.51)	6.95 (7.75–6.18)	12328.54 (13424.33–11401.89)	4.84 (5.27–4.47)	−1.29 (−1.47 to −1.12)
High-middle SDI	18950.04 (26233.54–14461.44)	6.9 (9.53–5.26)	13289.91 (17903.88–10668.16)	4.21 (5.66–3.38)	−1.45 (−1.59 to −1.31)
Middle SDI	30092.61 (44061.95–19386.38)	7.04 (10.38–4.55)	33119.4 (42624.17–24933.96)	5.25 (6.75–3.95)	−0.77 (−0.91 to −0.62)
Low-middle SDI	16953.39 (29026–8279.37)	6.48 (11.07–3.18)	29336.58 (41774.35–20702.66)	5.85 (8.32–4.13)	−0.26 (−0.36 to −0.16)
Low SDI	6287.57(15121.04–2217.15)	5.77 (13.89–2.07)	14975.82 (30263.79–8175.32)	5.5 (11.03–3.03)	−0.19 (−0.25 to −0.13)
GBD regions					
Andean Latin America	595.9 (944.79–364.5)	6.52 (10.27–4.03)	705.33 (1014.15–482.87)	4.03 (5.8–2.76)	−1.39 (−1.68 to −1.09)
Australasia	406.49 (556.81–299.99)	7.53 (10.33–5.56)	326.86 (416.05–255.5)	4.31 (5.48–3.36)	−2.05 (−2.56 to −1.54)
Caribbean	1046.16 (1530.45–709.37)	11.59 (16.81–7.91)	908.9 (1540.22–544.5)	7.55 (12.81–4.51)	−1.92 (−2.22 to −1.63)
Central Asia	2337.25 (3181.04–1514.61)	13.59 (18.48–8.79)	2896.21 (4226.43–1759.82)	11.81 (17.24–7.18)	−0.64 (−0.83 to −0.44)
Central Europe	2206.91 (2659.57–1819.57)	7.11 (8.58–5.87)	1539.1 (1821.51–1303.81)	5.62 (6.65–4.76)	−0.53 (−0.73 to −0.34)
Central Latin America	1341.74 (1719.88–1045.78)	3.42 (4.39–2.68)	1694.66 (2004.62–1431.59)	2.47 (2.92–2.08)	−1.5 (−1.67 to −1.32)
Central Sub-Saharan Africa	404.83 (1100.03–154.02)	3.42 (9.45–1.32)	1146.04 (2714.01–488.62)	3.57 (8.5–1.53)	0.23 (0.16 to 0.3)
East Asia	20937.1 (36489.09–11479.24)	6.54 (11.38–3.59)	14654.28 (23369.2–8667.35)	4.25 (6.81–2.49)	−0.89 (−1.24 to −0.54)
Eastern Europe	3373.6 (3990.03–2676.72)	6.11 (7.2–4.88)	1419.75 (1664.26–1207.12)	2.86 (3.36–2.43)	−2.82 (−3.2 to −2.44)
Eastern Sub-Saharan Africa	2555.61 (7798.86–864.58)	5.79 (17.93–1.98)	5608.89 (15815.74–2334.47)	5.05 (14.36–2.13)	−0.63 (−0.73 to −0.53)
High-income Asia Pacific	4606.61 (5128.06–4175.96)	9.97 (11.12–9.02)	2737.76 (3037.76–2457.09)	7 (7.77–6.29)	−1.28 (−1.37 to −1.19)
High-income North America	5457.47 (6188.17–4551.09)	7.13 (8.1–5.96)	4959.67 (5457.45–4493.77)	5.68 (6.25–5.14)	−1.05 (−1.28 to −0.81)
North Africa and Middle East	8841.13 (14025.97–4765.16)	11.66 (18.51–6.27)	12811.2 (18153.25–7535.45)	8.01 (11.35–4.71)	−0.93 (−1.02 to −0.84)
Oceania	125.76 (292.51–47.2)	8.39 (19.23–3.2)	331.38 (679.52–136.54)	9.5 (19.52–3.95)	0.35 (0.2 to 0.5)
South Asia	13893.03 (26113.16–5347.03)	5.73 (10.71–2.24)	26302.12 (38358.59–16412.7)	5.38 (7.83–3.36)	−0.04 (−0.15 to 0.07)
Southeast Asia	6362.8 (12075.1–3717.61)	5.49 (10.55–3.23)	9041.1 (15414.61–6268.54)	4.89 (8.31–3.38)	−0.32 (−0.41 to −0.24)
Southern Latin America	1754 (2298.64–1314.96)	14.26 (18.67–10.7)	1413.99 (1772.39–1127.14)	7.96 (9.99–6.33)	−1.87 (−2.03 to −1.71)
Southern Sub-Saharan Africa	435.99 (672.17–216.29)	3.29 (5.04–1.68)	650.56 (1001.56–350.4)	3.03 (4.65–1.63)	0.29 (−0.2 to 0.78)
Tropical Latin America	4022.43 (4486.6–3604.29)	10.51 (11.7–9.42)	5945.33 (6586.11–5374.69)	9.52 (10.55–8.6)	−0.84 (−1.25 to −0.43)
Western Europe	5764.09 (6966.44–4906.63)	5.98 (7.23–5.1)	3875.2 (4309.31–3504.4)	3.98 (4.42–3.59)	−1.42 (−1.92 to −0.92)
Western Sub-Saharan Africa	1917.93 (5267.45–733.43)	4.53 (12.58–1.76)	4183.01 (9065.78–1862.18)	3.45 (7.47–1.55)	−1.07 (−1.2 to −0.94)
(B)					
Global	1506.78 (2134.63–1036.78)	0.12 (0.17–0.08)	1777.8 (2388.86–1404.62)	0.09 (0,12–0.07)	−0.82 (−0.91 to −0.73)
SDI quintiles					
High SDI	280.94 (312.23–248.21)	0.12 (0.13–0.11)	218.85 (238.22–201.75)	0.08 (0.09–0.08)	−1.32 (−1.5 to −1.13)
High-middle SDI	322.78 (449.85–243.52)	0.12 (0.17–0.09)	231.96 (318.94–183.35)	0.07 (0.1–0.06)	−1.54 (−1.69 to −1.39)
Middle SDI	510.7 (759.47–324.03)	0.12 (0.18–0.08)	579.81 (756.68–430.86)	0.09 (0.12–0.07)	−0.83 (−0.97 to −0.69)
Low-middle SDI	287.42 (497.14–137.43)	0.11 (0.2–0.05)	501.4 (720.9–349.38)	0.1 (0.15–0.07)	−0.29 (−0.39 to −0.19)
Low SDI	103.22 (254.17–34.78)	0.1 (0.24–0.03)	244.03 (500.72–130.57)	0.09 (0.19–0.05)	−0.24 (−0.29 to −0.18)
GBD regions					
Andean Latin America	9.85 (15.75–5.86)	0.11 (0.18–0.07)	11.7 (17.2–7.82)	0.07 (0.1–0.04)	−1.46 (−1.77 to −1.14)
Australasia	7.04 (9.7–5.12)	0.13 (0.18–0.09)	5.75 (7.42–4.44)	0.07 (0.1–0.06)	−2.05 (−2.56 to −1.53)
Caribbean	17.93 (26.25–12.1)	0.2 (0.3–0.14)	15.66 (26.58–9.38)	0.13 (0.22–0.08)	−2.04 (−2.35 to −1.74)
Central Asia	38.35 (52.39–24.54)	0.23 (0.31–0.15)	49.89 (73.07–30)	0.2 (0.3–0.12)	−0.58 (−0.78 to −0.38)
Central Europe	38.8 (46.82–31.66)	0.12 (0.15–0.1)	27.94 (33.3–23.54)	0.1 (0.12–0.08)	−0.56 (−0.76 to −0.36)
Central Latin America	21.6 (28.25–16.55)	0.06 (0.07–0.04)	26.96 (32.36–22.52)	0.04 (0.05–0.03)	−1.7 (−1.89 to −1.51)
Central Sub-Saharan Africa	6.41 (18.5–2.19)	0.06 (0.17–0.02)	18.55 (45.6–7.41)	0.06 (0.15–0.02)	0.28 (0.2 to 0.35)
East Asia	358.67 (633.87–192.68)	0.12 (0.2–0.06)	259.79 (425.33–147.46)	0.07 (0.12–0.04)	−0.99 (−1.35 to −0.64)
Eastern Europe	55.97 (66.47–43.87)	0.1 (0.12–0.08)	23.53 (28–19.89)	0.05 (0.05–0.04)	−2.95 (−3.35 to −2.54)
Eastern Sub-Saharan Africa	40.31 (127.22–12.91)	0.1 (0.31–0.03)	88.12 (255.9–35.17)	0.08 (0.24–0.03)	−0.66 (−0.76 to −0.55)
High-income Asia Pacific	80.77 (89.74–73.42)	0.17 (0.19–0.16)	49.01 (54.52–43.89)	0.12 (0.13–0.11)	−1.29 (−1.38 to −1.2)
High-income North America	98.57 (112.12–81.6)	0.13 (0.15–0.11)	90.24 (99.29–81.53)	0.1 (0.11–0.09)	−1.07 (−1.31 to −0.83)
North Africa and Middle East	148.15 (237–78.53)	0.2 (0.33–0.11)	220.57 (316.32–127.95)	0.14 (0.2–0.08)	−0.98 (−1.08 to −0.88)
Oceania	2.12 (4.96–0.77)	0.15 (0.34–0.05)	5.57 (11.56–2.29)	0.16 (0.34–0.07)	0.25 (0.1 to 0.4)
South Asia	239.35 (454.18–89.24)	0.1 (0.19–0.04)	455.54 (668.72–278.01)	0.09 (0.14–0.06)	−0.09 (−0.19 to 0.01)
Southeast Asia	106.99 (208.51–61.27)	0.1 (0.19–0.05)	156.4 (273.94–106.57)	0.08 (0.15–0.06)	−0.36 (−0.43 to −0.28)
Southern Latin America	30.6 (40.17–22.8)	0.25 (0.33–0.19)	24.95 (31.48–19.8)	0.14 (0.18–0.11)	−1.9 (−2.07 to −1.73)
Southern Sub-Saharan Africa	6.93 (10.84–3.32)	0.05 (0.08–0.03)	10.74 (16.76–5.59)	0.05 (0.08–0.03)	0.37 (−0.13 to 0.87)
Tropical Latin America	69.66 (77.62–62.37)	0.19 (0.21–0.17)	106.69 (118.11–96.49)	0.17 (0.19–0.15)	−0.89 (−1.32 to −0.47)
Western Europe	98.07 (119.13–82.58)	0.1 (0.12–0.08)	66.23 (73.49–59.7)	0.07 (0.07–0.06)	−1.48 (−2.01 to −0.95)
Western Sub-Saharan Africa	30.63 (87.61–10.74)	0.08 (0.22–0.03)	63.97 (145.12–25.99)	0.05 (0.12–0.02)	−1.3 (−1.45 to −1.15)
(C)					
Global	30186.16 (20247.97–43203.69)	2.38 (1.60–3.40)	46630.38 (31117.64–67003.26)	2.35 (1.57–3.37)	−0.01 (−0.04 to 0.01)
SDI quintiles					
High SDI	6349.49 (4403.55–8951.99)	2.74 (1.89–3.86)	7092.44 (4927.68–9980.98)	2.71 (1.88–3.81)	−0.01 (−0.03 to 0.01)
High-middle SDI	7170.01 (4915.01–10163.74)	2.66 (1.82–3.77)	8883.13 (6032.72–12652.44)	2.66 (1.80–3.79)	0.12 (0.06 to 0.19)
Middle SDI	9359.13 (6121.42–13635.18)	2.27 (1.49–3.30)	15203.63 (10034.03–21927.11)	2.36 (1.56–3.41)	0.09 (0.04 to 0.14)
Low-middle SDI	4932.51 (3167.51–7323.81)	1.96 (1.26–2.90)	10101.19 (6530.62–14892.37)	2.05 (1.33–3.02)	0.16 (0.14 to 0.19)
Low SDI	2343.93 (1494.64–3483.72)	2.28 (1.46–3.39)	5312.58 (3381.49–7943.09)	2.10 (1.34–3.14)	−0.27 (−0.36 to −0.18)
GBD regions					
Andean Latin America	265.90 (175.37–387.17)	3.07 (2.03–4.46)	518.07 (342.13–750.58)	2.97 (1.96–4.30)	−0.23 (−0.33 to −0.14)
Australasia	165.34 (115.49–233.17)	3.07 (2.03–4.46)	225.09 (155.64–319.34)	2.97 (1.96–4.30)	−0.1 (−0.12 to −0.07)
Caribbean	240.54 (162.07–345.16)	3.07 (2.03–4.46)	307.93 (204.78–444.13)	2.97 (1.96–4.30)	−0.4 (−0.5 to −0.3)
Central Asia	240.54 (162.07–345.16)	2.67 (1.83–3.80)	307.93 (204.78–444.13)	2.58 (1.74–3.70)	−0.04 (−0.1 to 0.02)
Central Europe	844.73 (589.99–1177.37)	2.69 (1.88–3.75)	722.46 (493.59–1025.59)	2.58 (1.76–3.65)	−0.02 (−0.09 to 0.04)
Central Latin America	1153.70 (766.60–1660.81)	3.03 (2.02–4.35)	2371.96 (1613.85–3392.39)	3.44 (2.34–4.93)	0.13 (−0.03 to 0.29)
Central Sub-Saharan Africa	325.84 (209.46–484.47)	2.90 (1.87–4.31)	594.57 (373.49–891.58)	1.97 (1.24–2.94)	−1.03 (−1.17 to −0.88)
East Asia	7056.62 (4640.42–10189.27)	2.26 (1.49–3.27)	8610.85 (5735.02–12411.80)	2.35 (1.56–3.38)	0.2 (0.16 to 0.24)
Eastern Europe	1843.39 (1307.89–2563.82)	3.27 (2.32–4.55)	1617.13 (1126.16–2265.69)	3.05 (2.12–4.28)	−0.07 (−0.31 to 0.17)
Eastern Sub-Saharan Africa	1843.39 (1307.89–2563.82)	2.55 (1.63–3.79)	1617.13 (1126.16–2265.69)	2.17 (1.38–3.22)	−0.74 (−0.89 to −0.59)
High-income Asia Pacific	1470.91 (1050.50–2040.88)	3.12 (2.22–4.33)	1294.79 (922.50–1804.63)	3.02 (2.14–4.23)	−0.1 (−0.13 to −0.07)
High-income North America	1511.47 (1035.08–2145.34)	1.98 (1.35–2.81)	1649.74 (1110.81–2361.59)	1.88 (1.26–2.69)	−0.14 (−0.21 to −0.07)
North Africa and Middle East	1666.13 (1082.46–2447.04)	2.35 (1.53–3.45)	3641.67 (2389.06–5293.15)	2.27 (1.49–3.00)	−0.12 (−0.27 to 0.03)
Oceania	30.81 (19.72–45.75)	2.15 (1.38–3.19)	65.22 (41.16–98.60)	1.93 (1.22–2.93)	−0.04 (−0.15 to 0.06)
South Asia	4195.83 (2683.77–6255.59)	2.89 (1.88–4.24)	9042.59 (5794.78–13405.40)	1.77 (1.14–2.65)	0.18 (0.17 to 0.19)
Southeast Asia	2103.52 (1355.00–3121.29)	1.89 (1.22–2.81)	3567.27 (2292.37–5283.24)	1.90 (1.22–2.82)	0.05 (−0.07 to 0.16)
Southern Latin America	334.06 (230.17–473.37)	2.75 (1.89–3.89)	507.42 (354.15–711.99)	2.82 (1.96–3.95)	0.04 (0 to 0.08)
Southern Sub-Saharan Africa	239.87 (152.69–357.54)	1.96 (1.25–2.92)	444.70 (284.03–657.73)	2.07 (1.32–3.07)	0.07 (0.02 to 0.12)
Tropical Latin America	973.61 (646.39–1407.23)	2.60 (1.73–3.75)	1697.29 (1138.90–2436.28)	2.68 (1.80–3.85)	−0.01 (−0.16 to 0.15)
Western Europe	3307.76 (2284.03–4695.16)	3.38 (2.33–4.80)	3671.79 (2597.26–5123.81)	3.63 (2.56–5.07)	0.23 (0.2 to 0.25)
Western Sub-Saharan Africa	1043.18 (668.92–1550.65)	3.38 (2.33–4.80)	3302.33 (2154.21–4820.74)	3.63 (2.56–5.08)	0.72 (0.55 to 0.9)
(D)					
Global	5193.01(3210.049277–7828.797174)	0.41(0.252617577–0.617232985)	8532.89(5264.797718–12819.82759)	0.43(0.265563552–0.647111035)	0.12 (0.11 to 0.13)
SDI quintiles					
High SDI	667.02 (403.96–1021.61)	0.29 (0.17–0.44)	759.15 (459.28–1162.6)	0.29 (0.18–0.44)	−0.02 (−0.05 to 0.01)
High-middle SDI	877.77 (535.66–1338.84)	0.33 (0.2–0.5)	1132.59 (687.85–1724.52)	0.34 (0.2–0.51)	−0.05 (−0.12 to 0.02)
Middle SDI	1687.34 (1042.8–2534.82)	0.41 (0.25–0.62)	2655.18 (1633.9–4002.24)	0.41 (0.25–0.62)	0 (−0.04 to 0.04)
Low-middle SDI	1248.89 (777.72–1876.37)	0.5 (0.31–0.75)	2406.94 (1490.58–3591.46)	0.49 (0.3–0.73)	−0.15 (−0.18 to −0.12)
Low SDI	707.71 (447.1–1053.32)	0.69 (0.44–1.03)	1572.54 (986.85–2342.33)	0.63 (0.39–0.93)	−0.32 (−0.39 to −0.26)
GBD regions					
Andean Latin America	38.74 (23.62–58.67)	0.45 (0.27–0.67)	80.73 (49.67–122.06)	0.46 (0.29–0.7)	0.18 (0.1 to 0.27)
Australasia	15.34 (9.33–23.73)	0.28 (0.17–0.44)	22.73 (13.63–35.28)	0.3 (0.18–0.46)	0.12 (0.09 to 0.15)
Caribbean	33.25 (20.25–50.41)	0.38 (0.23–0.58)	50.41 (30.91–76.31)	0.41 (0.25–0.63)	0.38 (0.29 to 0.46)
Central Asia	47.77 (28.9–73.19)	0.31 (0.19–0.48)	82.49 (50.28–126.79)	0.33 (0.2–0.51)	0.09 (−0.01 to 0.18)
Central Europe	84.29 (50.51–129.14)	0.27 (0.16–0.41)	89.04 (53.54–135.73)	0.3 (0.18–0.46)	0.32 (0.23 to 0.42)
Central Latin America	172.01 (106.23–260.61)	0.45 (0.28–0.69)	282.47 (171.05–428.84)	0.41 (0.25–0.62)	−0.11 (−0.25 to 0.03)
Central Sub-Saharan Africa	79.04 (49.53–118.39)	0.71 (0.44–1.06)	209.24 (131.58–314.11)	0.7 (0.44–1.05)	0.19 (0.03 to 0.36)
East Asia	1135.37 (697.65–1716.56)	0.37 (0.23–0.56)	1307.52 (799.18–1967.44)	0.35 (0.22–0.53)	−0.2 (−0.23 to −0.16)
Eastern Europe	160.24 (96.11–248.66)	0.28 (0.17–0.44)	169.65 (102.41–260.09)	0.31 (0.19–0.48)	0.13 (−0.1 to 0.36)
Eastern Sub-Saharan Africa	340.28 (215.9–505.14)	0.88 (0.56–1.31)	786 (497.77–1163.6)	0.81 (0.51–1.2)	−0.12 (−0.2 to −0.04)
High-income Asia Pacific	116.95 (69.69–180.88)	0.25 (0.15–0.38)	111.39 (66.35–171.84)	0.26 (0.16–0.4)	0.11 (0.07 to 0.14)
High-income North America	167.84 (101.18–259.55)	0.22 (0.13–0.34)	213.02 (129.13–327.54)	0.24 (0.15–0.37)	0.3 (0.22 to 0.38)
North Africa and Middle East	313.37 (193.24–476.36)	0.45 (0.27–0.68)	656.19 (404.15–995.55)	0.41 (0.25–0.62)	−0.44 (−0.58 to −0.31)
Oceania	6.58 (4.03–9.87)	0.47 (0.29–0.7)	16.84 (10.38–25.51)	0.5 (0.31–0.76)	0.02 (−0.06 to 0.1)
South Asia	1109.44 (693.25–1663.17)	0.47 (0.29–0.7)	2257.42 (1397.5–3371.51)	0.47 (0.29–0.7)	−0.07 (−0.1 to −0.04)
Southeast Asia	458.27 (284.27–689.36)	0.42 (0.26–0.63)	800.76 (493.14–1203.36)	0.43 (0.26–0.64)	−0.02 (−0.09 to 0.05)
Southern Latin America	32.83 (19.49–51.16)	0.27 (0.16–0.42)	47.98 (28.6–74.31)	0.27 (0.16–0.41)	0 (−0.03 to 0.03)
Southern Sub-Saharan Africa	88.22 (55.77–130.6)	0.73 (0.46–1.08)	143.23 (89.85–210.44)	0.67 (0.42–0.98)	−0.1 (−0.2 to −0.01)
Tropical Latin America	145.58 (88.55–220.25)	0.39 (0.24–0.59)	244.43 (149.03–368.96)	0.39 (0.24–0.58)	0.02 (−0.1 to 0.14)
Western Europe	357.11 (217.64–544.93)	0.37 (0.22–0.56)	334.38 (201.72–515.51)	0.33 (0.2–0.51)	−0.38 (−0.44 to −0.31)
Western Sub-Saharan Africa	290.49 (183.73–431.25)	0.75 (0.47–1.11)	626.97 (389.76–939.81)	0.58 (0.36–0.87)	−1.11 (−1.23 to −0.99)

ASR, age-standardized rate; PAH, pulmonary arterial hypertension; SDI, sociodemographic index; EAPC, estimated annual percentage change; UIs, uncertainty intervals; CI, confidence interval.

**Figure 4:**
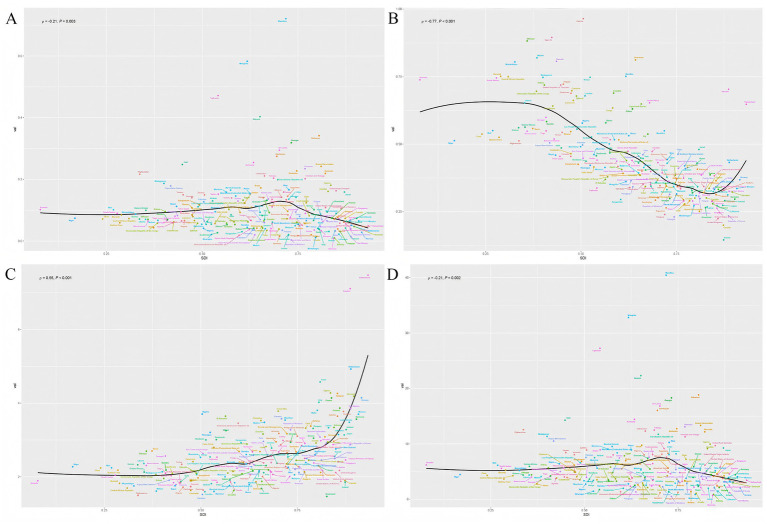
The relationship between the social demographic index (SDI) and the ASR of deaths, incidence, prevalence, and DALYs in different countries. **(A)** Deaths. **(B)** Incidence. **(C)** Prevalence. **(D)** DALYs. The ρ value and *p* value in the upper left corner of each subgraph represent the correlation coefficient and the statistical significance of the relationship, respectively.

At the national level, according to Supplementary Table 1 and Figure 4, Mauritius [ASMR (95%UIs): 0.72 (0.51–0.97)], Mongolia [ASMR (95%UIs): 0.58 (0.06–0.95)], and Tajikistan [ASMR (95%UIs): 0.47 (0.10–0.87)] were the top 3 countries with the highest ASR values of deaths for PAH in 2021 (Figure 4A). Moreover, Zambia [ASIR (95%UIs): 0.96 (0.61–1.41)], Uganda [ASIR (95%UIs): 0.89 (0.57–1.33)], and Ethiopia [ASIR (95%UIs): 0.88 (0.56–1.29)] were the were the top 3 countries with the highest ASR values of incidence for PAH in 2021 (Figure 4B). Switzerland [ASPR (95%UIs):7.47 (5.32–10.39)], Sweden [ASPR (95%UIs): 7.11 (4.96–9.98)], and Netherlands [ASPR (95%UIs): 4.93 (3.41–6.98)] were the top 3 countries with the highest ASR values of prevalence for PAH in 2021 (Figure 4C). Mauritius [ASDR (95%UIs): 9.95 (3.61–17.75)], Mongolia [ASDR (95%UIs): 9.75 (8.81–10.81)], and Tajikistan [ASDR (95%UIs): 9.68 (3.06–16.91)] were the top 3 countries with the highest ASR values of DALYs for PAH in 2021 (Figure 4D).

### Health inequalities in PAH among WCBA

**Figure 5:**
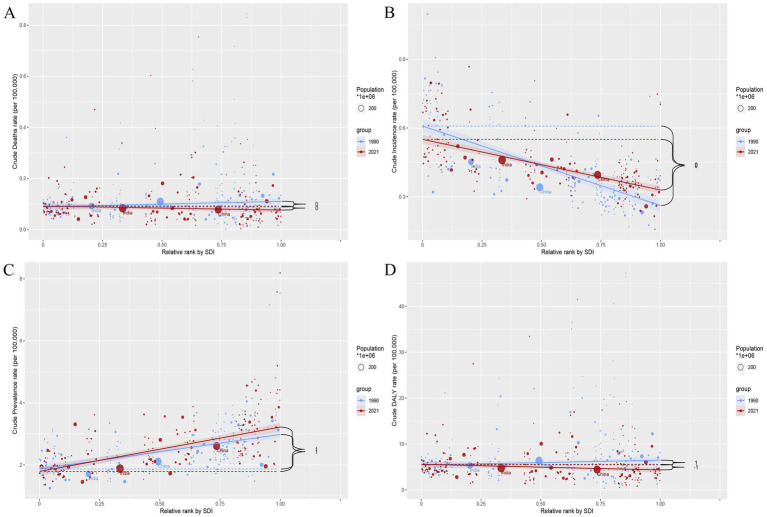
Slope index of inequality (SII) for deaths, incidence, prevalence, and DALYs by SDI in 1990 and 2021. **(A)** Deaths. **(B)** Incidence. **(C)** Prevalence. **(D)** DALYs. The blue and red dashed lines represent data for 1990 and 2021, respectively. The shaded area indicates the 95% confidence interval for the slope index of inequality.

**Figure 6:**
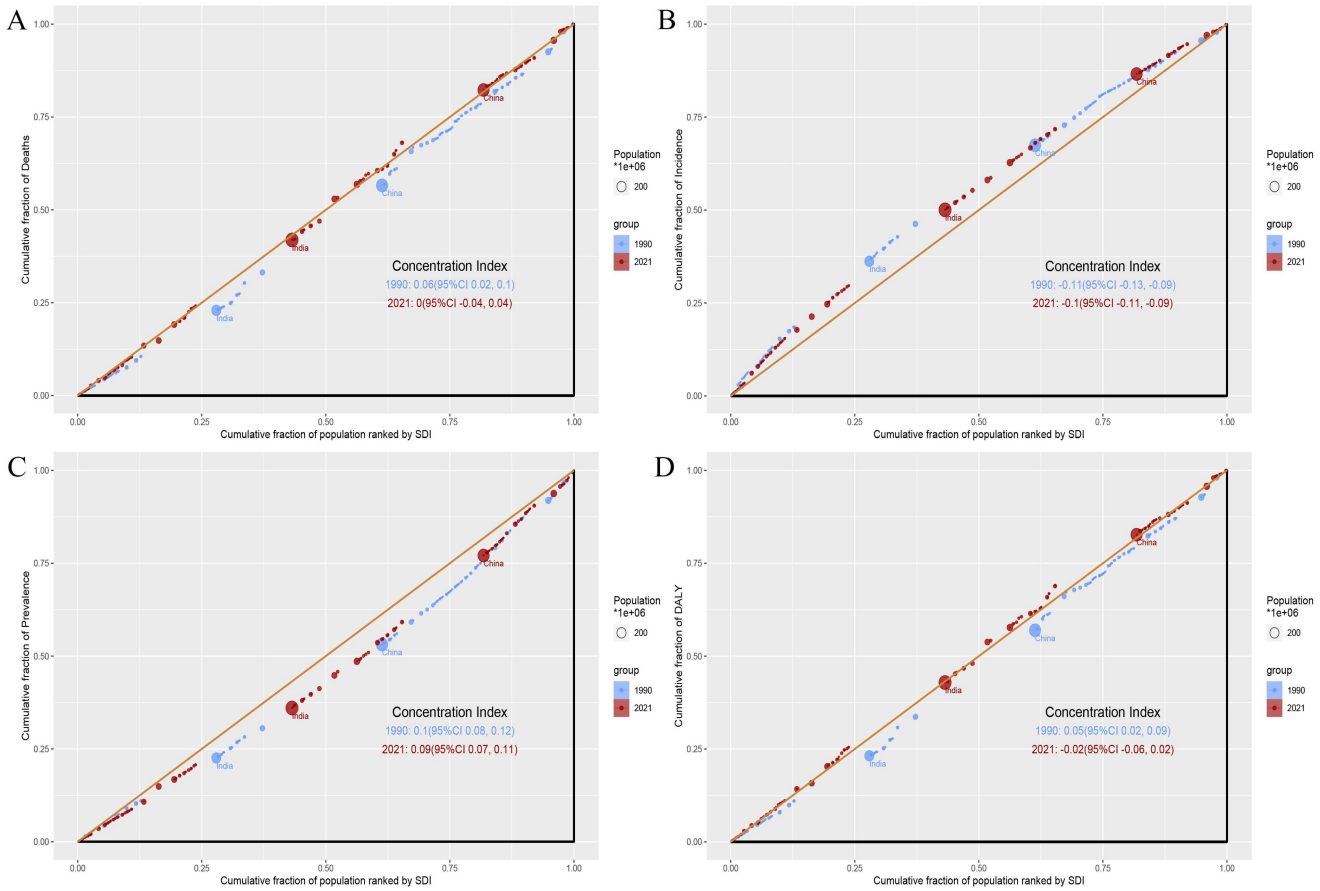
Concentration index (CI) for deaths, incidence, prevalence, and DALYs by SDI in 1990 and 2021. **(A)** Deaths. **(B)** Incidence. **(C)** Prevalence. **(D)** DALYs. Blue dots and lines represent data from 1990, while red dots and lines represent data from 2021. Larger circles indicate data for populations of 1 million or more.

To further investigate health inequalities in PAH among WCBA, we conducted studies on the slope index and concentration index (Figures 5, 6). The slope index results showed that incidence decreased as the SDI increased, and compared to 1990, incidence decreased in all regions by 2021. In contrast, the prevalence increases as the SDI rises, and the prevalence in 2021 increased across all regions. However, there were no significant trends in deaths or DALYs (Figures 5A,D). The concentration index results were similar, with incidence data points predominantly distributed in low SDI region and prevalence data points predominantly distributed in high SDI region, and the absolute values of both concentration index remained largely unchanged (Figures 6B,C). Notably, the concentration index for DALYs showed a relatively large change, indicating a slight decrease in concentration (Figure 6D).

### Overall trends in PAH burden by using EAPC at global and regional levels

Globally, from 1990 to 2021, the EAPC (95% CI) values for deaths, incidence, prevalence, and DALYs in PAH were −0.82 (−0.91 to −0.73), 0.12 (0.11 to 0.13), −0.01 (−0.04 to 0.01), and −0.76 (−0.84 to −0.67) per year, respectively (Tables 1A–D; Supplementary Table 2). For the five SDI regions, nearly all EAPC (95% CI) values for deaths, incidence, and DALYs in PAH exhibited a downward trend from 1990 to 2021, with the exception of prevalence. Specifically, the largest absolute changes in EAPC (95% CI) values for deaths and DALYs occurred in the high-middle SDI region, at −1.54 (−1.69 to −1.39) and −1.45 (−1.59 to −1.31), respectively (Tables 1A,D; Supplementary Table 2). The largest absolute changes in EAPC (95% CI) values for incidence and prevalence were observed in the low SDI region, at −0.32 (−0.39 to −0.26) and −0.27 (−0.36 to −0.18), respectively (Tables 1B,C; Supplementary Table 2).

### Joinpoint regression analysis on local trends in PAH burden

**Figure 7:**
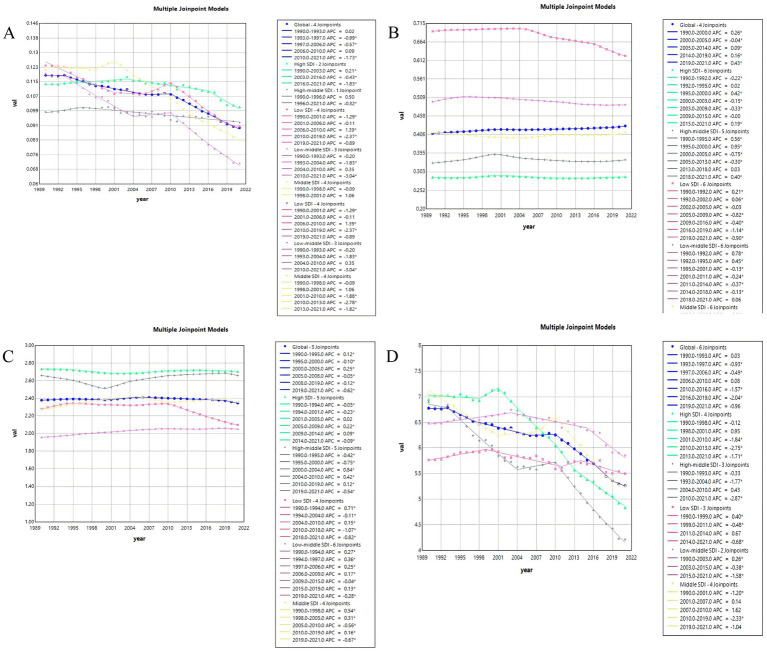
Joinpoint regression analysis on the ASR of deaths, DALYs, prevalence, and incidence with PAH in global and 5 SDI regions. **(A)** Deaths. **(B)** Incidence. **(C)**Prevalence. **(D)** DALYs.

Between 1990 and 2021, the global ASR values of incidence (AAPC = 0.16) and prevalence (AAPC = −0.05) of PAH slightly changed. However, the ASR values of deaths (AAPC = −0.82) and DALYs (AAPC = −0.90) for PAH showed an initial increase from 1990 to 1993, followed by a decrease from 1993 to 2006, a slight increase from 2006 to 2010, and finally a decline from 2010 to the lowest point in 2021. Similarly, the five SDI regions also showed an overall downward trend in the ASR values of deaths and DALYs, with a sharp decline beginning in 2010 and reaching its lowest point in 2021 (Figures 7A,D). This clearly shows that after 2010, the disease burden of PAH in terms of deaths and DALYs among WCBA has been greatly reduced, both globally and in the five SDI regions. However, the trend in the five SDI region differs from the global trend in terms of incidence and prevalence (Figures 7B,C). The ASR values of incidence in low SDI region exhibited a significant downward trend from 2016 to 2019(APC = −1.14) and 2019 to 2021(APC = −0.90), as did ASR values of prevalence 2010 to 2018(APC = −1.07) and 2018 to 2021(APC = −0.82).

### Decomposition analysis on PAH burden in 2021

**Figure 8:**
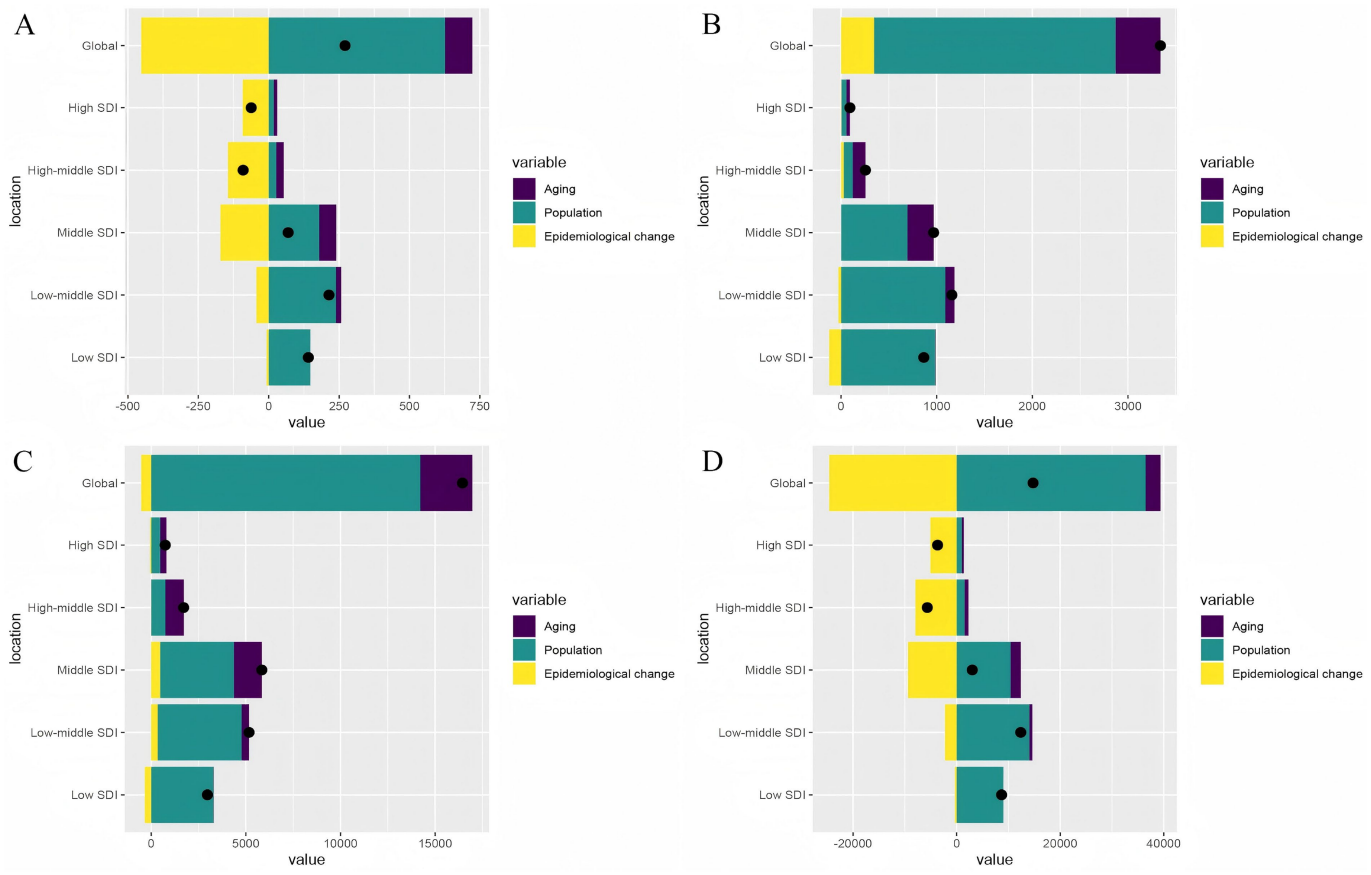
Decomposition analysis on the ASR of deaths, DALYs, prevalence, and incidence with PAH in global and 5 SDI regions. **(A)** Deaths. **(B)** Incidence. **(C)**Prevalence. **(D)** DALYs.

We conducted a decomposition analysis of deaths, prevalence, incidence, and DALYs, both globally and across five SDI regions, to investigate the impacts of population growth, aging, and epidemiologic changes on the burden of PAH during the study period. The results are presented in Figure 8 and Supplementary Table 10. Black dots indicate the overall change caused by all three components. For individual components, a positive magnitude signifies a corresponding increase, while negative magnitudes signify a decrease. Globally, there was a significant increase in the age-standardized rates (ASR) of deaths, DALYs, incidence, and prevalence, with population growth as the largest modifier (Figures 8A–D). Among the five SDI regions, the low-medium SDI exhibited the highest increase in deaths, incidence, and DALYs (Figures 8A–D). In terms of prevalence, the largest increase was observed in the low SDI region (Figure 8C). Overall, population growth was the main driver globally, accounting for 231.02, 247.17, 75.63, and 86.46% of the increased burden of deaths, DALYs, incidence, and prevalence, respectively, from 1990 to 2021 (Supplementary Table 10). Similar to the global trend, population growth was the primary driving factor for the comprehensive increase in burden in the low SDI, low-medium SDI, and medium SDI regions. However, in the other two regions, epidemiological changes were the main factors driving the growth of ASR values of deaths and DALYs in the high SDI region (deaths: 148.45%, DALYs: 137.83%; Figures 8A,D; Supplementary Table 10), whereas aging was the main driver for the growth of ASR values of incidence and prevalence in the high-middle SDI region (prevalence: 56.76%, incidence: 52.28%; Figures 8B,C; Supplementary Table 10). It is concerning that epidemiological changes were either negative or not significant contributors to positive increases in deaths, DALYs, incidence, and prevalence in the low SDI, low-middle SDI, and middle SDI regions.

### Predictive analysis on PAH trend to 2040

**Figure 9:**
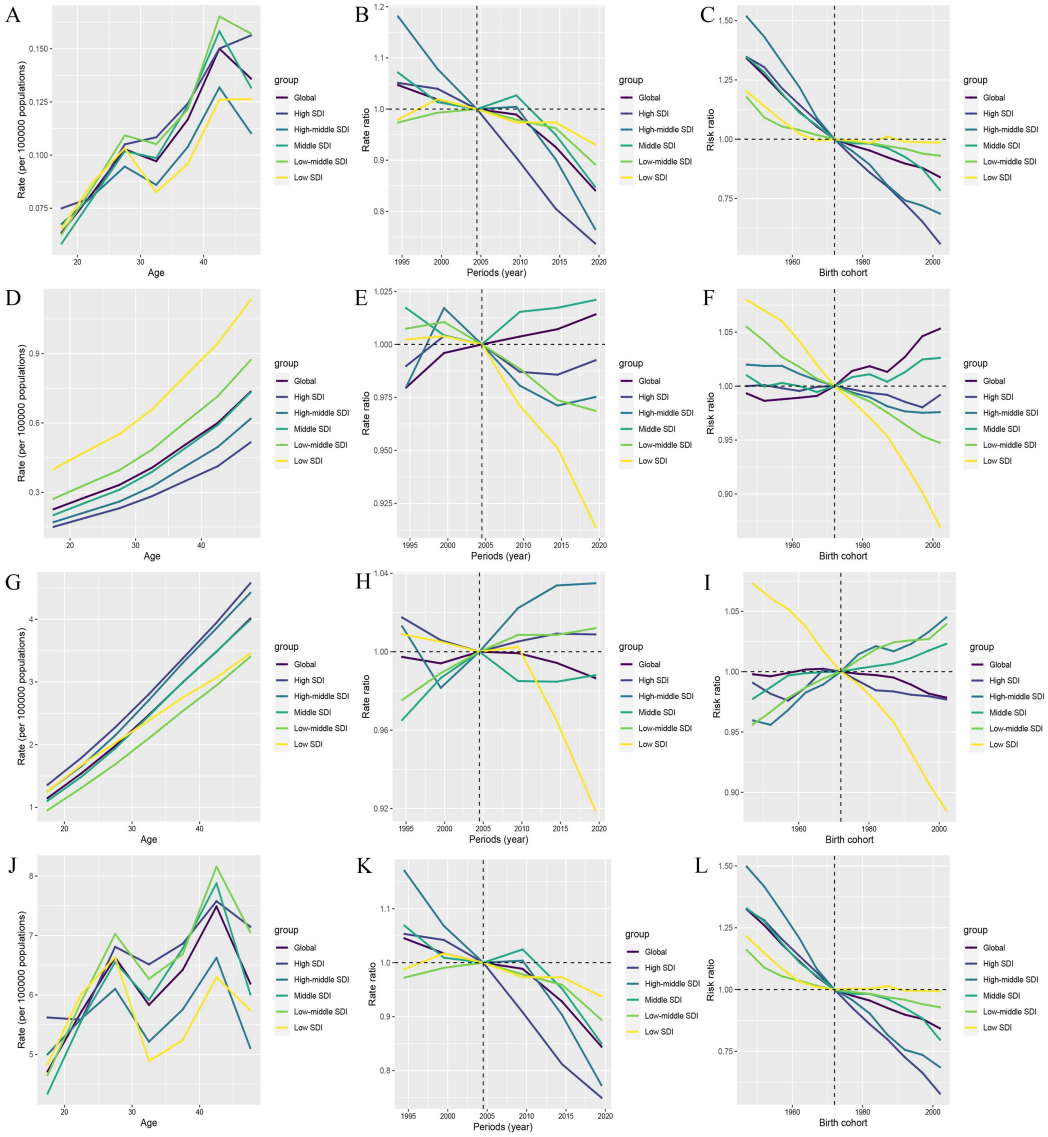
The impact of age, period, and birth cohort on PAH burden among WCBA, based on the age-period-cohort (APC) model. **(A–C)** Deaths. **(D–F)** Incidence. **(G–I)** Prevalence. **(J–L)** DALYs.

**Figure 10:**
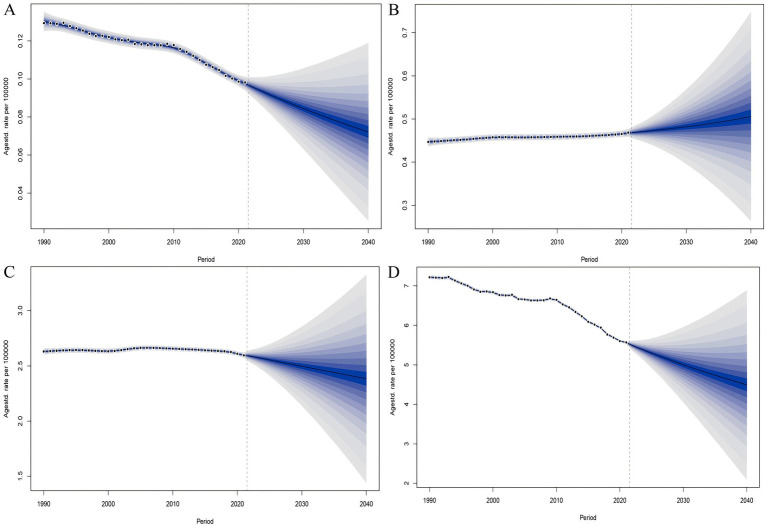
The BAPC model predicted the disease burden of PAH in the WCBA from 2022 to 2040. **(A)** Deaths. **(B)** Incidence. **(C)** Prevalence. **(D)** DALYs.

**Figure 11:**
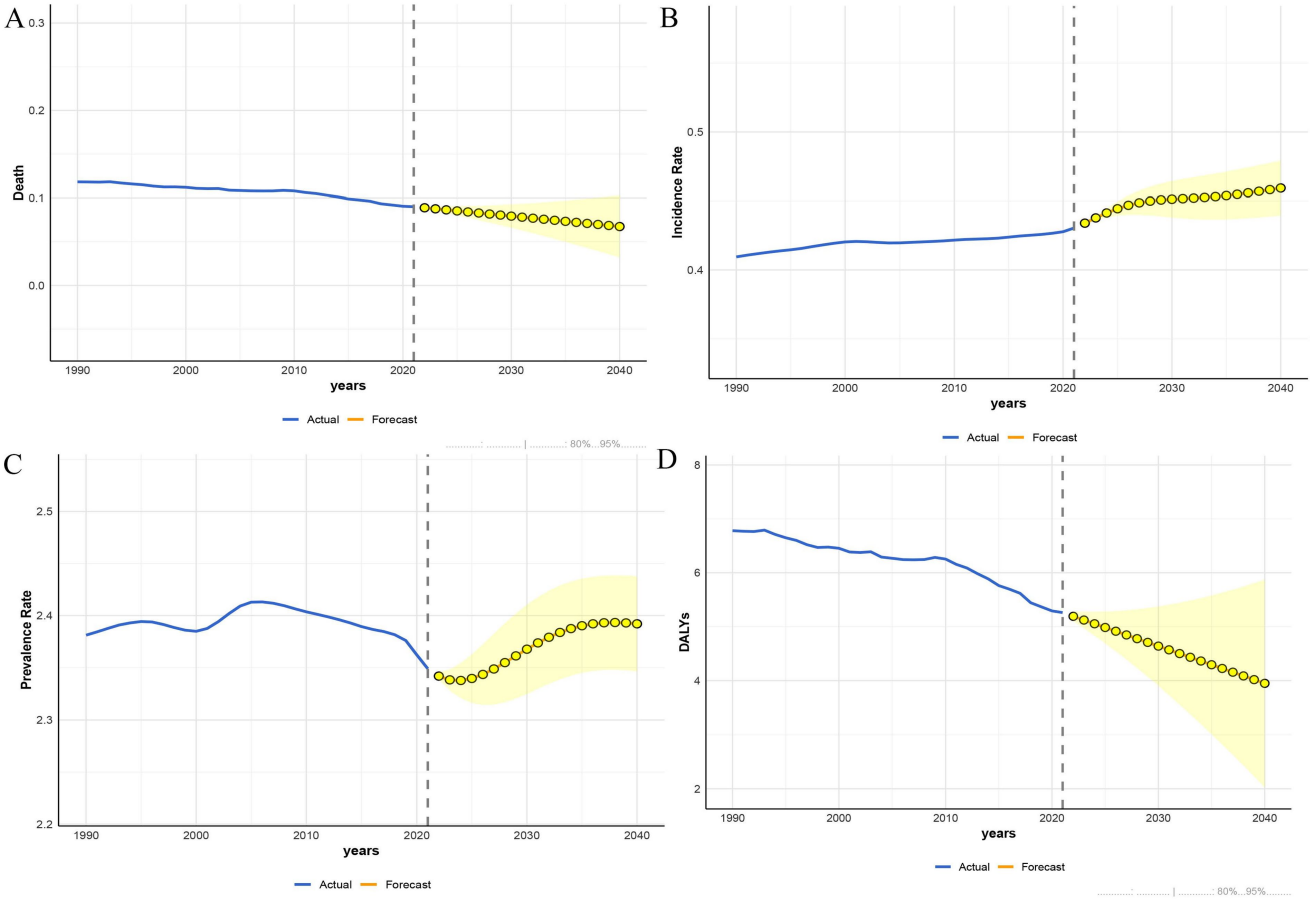
The ARIMA model predicted the disease burden of PAH in the WCBA from 2022 to 2040. **(A)** Deaths. **(B)** Incidence. **(C)** Prevalence. **(D)** DALYs.

**Figure 12:**
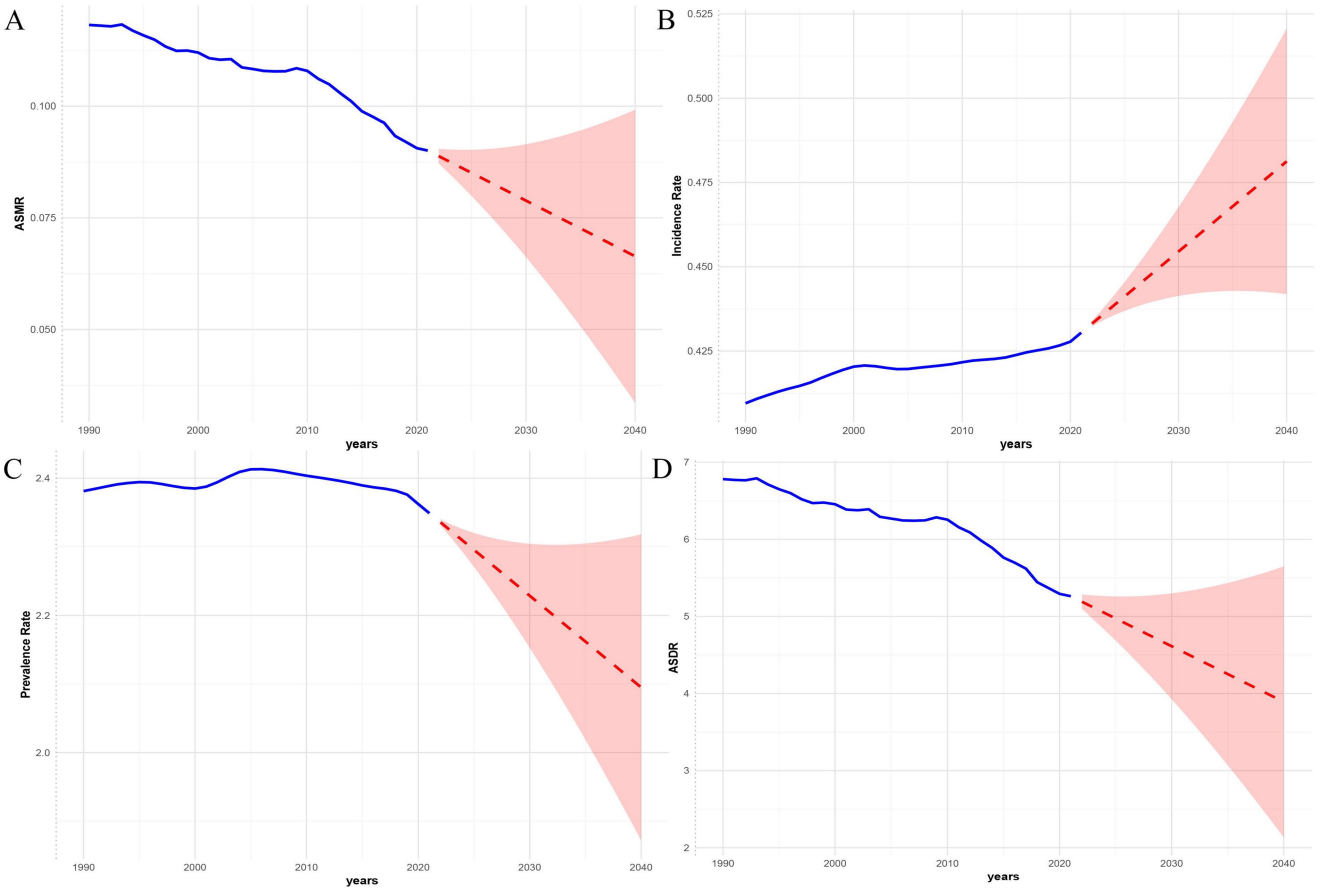
The ETS model predicted the disease burden of PAH in the WCBA from 2022 to 2040. **(A)** Deaths. **(B)** Incidence. **(C)** Prevalence. **(D)** DALYs.

Finally, based on the APC analysis, we used BAPC, ARIMA, and ETS models to predict future trends of PAH in WCBA from 2022 to 2040 globally. Details of the APC analysis are shown in Figure 9, and the prediction results are shown in Figures 10–12 and Supplementary Tables 11–13. All of the three models predict that ASMR and ASDR are projected to descend globally in the coming years (Figures 10–12). The BAPC model indicated that the indexes (RMSE, MAE, MAPE) in the BAPC model were better than those of the other models when predicting ASMR, ASDR and prevalence rate (Supplementary Tables 11–13). The ETS was better than those of the other models when predicting incidence rate. According to the best preformed model, global ASMR, ASDR, prevalence rate and incidence rate are expected to reach 0.07, 4.49, 2.39 and 0.46, respectively, by 2040 (Supplementary Tables 11–13).

## Discussion

This GBD study first quantified the burden of PAH among WCBA from 1990 to 2021 at global, five SDI regions, 204 countries and territories levels, and projected trends to 2040 using three models. The study results show a complex picture: while the absolute number of cases increased, the annual values for ASMR and ASDR are decreasing, indicating improvements in mortality outcomes. However, it is important to note that the ASIR for PAH in the global WCBA has been rising, signaling a persistent influx of new cases. Similarly, the results of the joint point regression analysis are consistent with the EAPC. Fortunately, both globally and across the five SDI regions, there is a noticeable significant reduction in PAH mortality and DALY burden. This is particularly evident in low-SDI regions, where the burden of PAH in the WCBA population decreased significantly during the periods from 2016 to 2021 and from 2020 to 2021. Decomposition analysis identified population growth as the primary driver of the increase in the PAH burden among WCBA populations globally, particularly in less developed regions, while epidemiological changes contributed to burden reduction in high-SDI areas. Multi-model predictive modeling results indicate that, with the support of improvements in healthcare and social welfare, the PAH burden among the global WCBA population will continue to decline until 2040 in terms of mortality and DALYs, except for a potential continued rise in incidence rates. However, population growth, aging, and uneven development will continue to pose ongoing challenges to global PAH control.

The GBD study found similar estimates of 2021 global PAH incidence and prevalence as previous related research (incidence 0.43/100,000 person-years vs. 0.4/100,000 person-years, prevalence 2.35/100,000 vs. 3/100,000) (12). The updated 2021 global deaths and DALYs are also reported (deaths 0.09/100,000, DALYs 5.26/100,000), contributing significantly to global epidemiological research. Additionally, although the number of deaths, incidence, prevalence, and DALY cases for PAH among WCBA indicated an upward trend, their ASR values showed a downward trend between 1990 and 2021. This change in incidence may be associated with progress in the prevention and control of PAH-related diseases such as hemodialysis, schistosomiasis, HIV infection, metabolic syndrome, and sickle cell disease in developing countries (34–36). Furthermore, improvements in global cardiovascular risk management (37–39) are vital reasons for the decline in the growth of PAH incidence among WCBA. The decrease in ASR values for deaths and DALYs may primarily stem from the widespread application of multi-drug combination therapies (combined with the efficacy of phosphodiesterase type-V inhibitors (PDE-Vi), endothelin type-A, and type-Vi) (38), new drugs [including endothelin type-A and type-B receptor antagonists (ERAs), prostaglandin I2, and the GRIPHON trial with selexipag (a prostaglandin I2 receptor agonist)] (40–42), and advances in basic research on PAH-related biomarkers (HGFA) (43). However, the accelerated increase in incidence (EAPC > 0) highlights the ongoing influx of new cases of PAH among WCBA, posing a significant challenge to global health systems and economies.

In addition, we discovered that the burden of PAH among WCBA exhibits a significant SDI gradient. In 2021, the ASR values for deaths, incidence, and DALYs of PAH among WCBA were highest in low-middle SDI regions, reflecting greater challenges in controlling new cases due to limited medical resources. These disparities likely reflect a combination of true higher incidence due to greater exposure to risk factors (e.g., untreated infections, environmental exposures) and differentials in diagnosis and care access. In low SDI regions, underreporting and data underestimation, limited diagnostic capabilities, and an incomplete disease surveillance system may primarily account for the lower ASR values compared to low-middle SDI regions. Interestingly, the prevalence of PAH among WCBA was highest in high SDI regions in 2021 and decreased as SDI levels declined. This suggests that a significant number of PAH patients achieve good outcomes after treatment, but the accumulation of existing patients will result in ongoing economic and health burdens (44). It is evident that the core of health inequalities in PAH among WCBA lies in the disparities in the risks they face and their access to quality healthcare. According to previously published European and North American guidelines and consensus statements, high-burden regions with high fertility rates and limited medical resources should integrate PAH screening into maternal health care programs while comprehensively considering the broad etiology of the disease (45–47). In high SDI regions, due to the long-term exposure risk associated with population aging, long-term management measures should be optimized (48, 49), such as establishing a long-term follow-up drug management system for older populations of WCBA.

When dividing the trends of the past three decades into sub-segments, Joinpoint regression revealed a significant finding: the sustained decline in ASR of deaths and DALYs due to PAH was followed by a transient increase from 2006 to 2010 globally, after which there was a sharp decline from the 2010 joinpoint to the lowest point in 2021. This trend also impacted the five SDI regions. Regarding deaths, low SDI and low-middle SDI regions experienced a rebound between 2006 and 2010 (low SDI region: APC = 1.39, low-middle SDI region: APC = 0.35). In terms of DALYs, middle SDI and middle-high SDI regions exhibited rebounds from 2007 to 2010 and from 2004 to 2010, respectively (middle SDI region: APC = 1.62, middle-high SDI region: APC = 0.48). Furthermore, all five SDI regions also faced a sharp decline after the turning point in 2010, reaching their lowest levels by 2021. This fluctuating trend may be associated with multiple contextual factors, including economic shocks, accelerating urbanization, and changing lifestyle risks, though causal inferences cannot be definitively drawn from our data. Firstly, urbanization, which has accelerated in most developing countries since 2005, may be associated with increased exposure to environmental pollutants and occupational hazards linked to lung diseases, potentially influencing PAH risk (50). Secondly, rising obesity rates may be linked to an increased risk of cardiovascular disease, thereby potentially contributing to the related PAH burden (51). Lastly, the severe financial crisis between 2006 and 2010 may have negatively impacted health system capacities to prevent and treat PAH in some settings, potentially contributing to the observed increase in DALYs and deaths associated with PAH during that period (52). Meanwhile, we also noted that this rebound point influenced the process of PAH burden reduction, and it is essential to acknowledge that all PAH burdens have further declined in recent years post-2019, indicating that the latest global PAH control measures are effective and efficient.

Our decomposition analyses suggest that population growth is the primary driver of the increasing burden of PAH globally and in low- and middle-income regions (low-income/low-middle-income), particularly concerning incidence and prevalence. In contrast, population aging significantly impacts all indicators (including mortality and DALYs) in high-income regions. Notably, favorable epidemiological changes, such as improved management, were observed in high and high-middle SDI regions, particularly regarding reduced mortality and DALYs. The substantial role played by population growth in areas with low levels of development highlights the possible correlation between PAH risk, high fertility rates, and maternal and child health challenges in the WCBA population. This may be due to high fertility rates in low SDI regions expanding the base population of WCBA groups (53, 54), indirectly increasing the PAH burden on these groups. Conversely, the predominance of population aging in high SDI areas reveals emerging risks associated with delayed childbearing and longer exposure to PAH risk factors in this population. While the mitigating effects of epidemiological changes are encouraging, they appear to be unequal across SDI strata, likely reflecting disparities in healthcare access, diagnostic capabilities, and treatment availability.

Multi-model projections indicate that the ASIR for PAH cases in WCBA will rise globally by 2040, aligning with previous studies. However, in the future, the ASDR and ASMR for PAH in WCBA are expected to decline. This can be attributed to several factors. A potential reason is the global advancement in healthcare policies and the field of obstetrics and gynecology, which may lead to improved management and control of the disease, particularly in low SDI countries, potentially enhancing the prognosis of PAH in WCBA. Additionally, global economic growth may bolster healthcare access for the population, thereby contributing to reductions in ASDR and ASMR (55). The anticipated rise in future incidence may be linked to the expansion of the WCBA population base due to global population growth. Furthermore, disparities in PAH burden between gender subgroups reveal that women in areas with lower SDI bear a higher PAH burden. This divergent future trend in incidence and prognosis necessitates that our research and healthcare policies place greater emphasis on female PAH in the coming years.

The GBD database has some limitations. Firstly, the accuracy of the results of this study depends on the quality of the raw data from the GBD study. But the GBD study lacks population data from remote areas, and different diagnostic criteria may bias the analyses (56). This geographical and diagnostic inconsistency may distort our understanding of the actual impact of the disease. Secondly, although the GBD database can suggest future research directions, it does not address basic research, and further validation and extension of the mechanisms of PAH pathology is needed. Third, the GBD study did not distinguish between secondary and primary pulmonary hypertension. And GBD 2021 did not provide risk factors for PAH like other diseases. Due to the lack of categorical information, study results and healthcare strategies may not be able to make the best judgment based on the specific type of PAH, which affects the applicability of the study in actual clinical settings (57). Fourth, registry data in many countries, particularly low-SDI ones, are incomplete or non-existent, and misdiagnosis or under-diagnosis of PAH is common, especially where diagnostic facilities like right heart catheterization are scarce. This could lead to significant underestimation of the true burden in these regions. Fifth, our predictive models, while robust, inherit the uncertainties of the input GBD data. Although we propagated uncertainty intervals into our projections, these models lack real-world clinical validation and cannot account for unforeseen changes in healthcare systems or the introduction of novel therapies. Sixth, the absence of treatment-level data in GBD limits our ability to directly attribute burden changes to specific interventions or health system factors. Finally, the ecological nature of the study means that observed associations between trends and potential drivers should be interpreted as correlational rather than causal. We hope that in the future, the GBD database will continue to improve, making the study country comprehensive and complete.

### Policy implications

Based on our findings, we propose the following concrete policy actions tailored to different SDI contexts:

For Low and Low-Middle SDI Regions: Integrate simple, cost-effective PAH screening into routine antenatal care and for women with known risk factors within primary health care systems. Implement task-shifting by training non-physician healthcare workers in the initial recognition of suspected PAH. Develop and finance dedicated referral pathways for confirmed cases to access specialist care. Advocate for the inclusion of essential PAH medications in national health insurance schemes.

For Middle and High-Middle SDI Regions: Strengthen regional diagnostic centers with capacity for right heart catheterization. Establish and fund national PAH registries to monitor disease burden, treatment patterns, and outcomes. Develop and disseminate national guidelines adapted to local resources and drug availability. Promote public and healthcare professional awareness campaigns about PAH symptoms and risk factors.

For High SDI Regions: Optimize long-term management by establishing multidisciplinary PAH-pregnancy clinics and structured transition programs for adolescents with PAH moving to adult care. Implement robust electronic health record systems to facilitate long-term follow-up and drug adherence monitoring. Focus research on the specific needs of the aging WCBA population with PAH.

Globally: Encourage international collaboration and funding mechanisms to support PAH care in resource-limited settings. WHO and other global health agencies should consider developing a technical package for PAH detection and management, similar to those for other non-communicable diseases.

## Conclusion

In summary, this GBD study provides a comprehensive overview of PAH deaths, incidence, prevalence, and DALYs among WCBA globally, across the five SDI regions, and in 204 countries and territories from 1990 to 2021, while also presenting projected analyses through 2040. From 1990 to 2021, the global burden of PAH among WCBA has generally increased but is now trending downward. The year 2010 marked a significant turning point in the considerable burden of PAH, with population aging and growth as the primary contributing factors. These results underscore the substantial challenge of controlling and managing PAH in WCBA. Moving forward, with an abundance of medical resources and the ongoing optimization of public health policies, the burden of PAH in WCBA is expected to trend toward decreasing deaths, reduced DALYs, and increasing morbidity. Each region and country should develop health policies that are locally appropriate to address diverse healthcare needs.

## Data Availability

The original contributions presented in the study are included in the article/Supplementary material, further inquiries can be directed to the corresponding author.
